# Controlled-release of apatinib for targeted inhibition of osteosarcoma by supramolecular nanovalve-modified mesoporous silica

**DOI:** 10.3389/fbioe.2023.1135655

**Published:** 2023-02-16

**Authors:** Xinglong Wang, Gongke Li, Ke Li, Yu Shi, Wenzheng Lin, Chun Pan, Dandan Li, Hao Chen, Jianwei Du, Huihui Wang

**Affiliations:** ^1^ Department of Orthopedics, Affiliated Hospital of Yangzhou University, Yangzhou, Jiangsu, China; ^2^ Department of Critical Care Medicine, Affiliated Hospital of Yangzhou University, Yangzhou, Jiangsu, China

**Keywords:** supramolecular nanovalves, HMSNs, Cd, AlN, APA

## Abstract

Targeted delivery of antitumor drugs has been recognized as a promising therapeutic modality to improve treatment efficacy, reduce the toxic side effects and inhibit tumor recurrence. In this study, based on the high biocompatibility, large specific surface area, and easy surface modification of small-sized hollow mesoporous silica nanoparticles β-cyclodextrin (β-CD)-benzimidazole (BM) supramolecular nanovalve, together with bone-targeted alendronate sodium (ALN) were constructed on the surface of small-sized HMSNs. The drug loading capacity and efficiency of apatinib (Apa) in HMSNs/BM-Apa-CD-PEG-ALN (HACA) were 65% and 25%, respectively. More importantly, HACA nanoparticles can release the antitumor drug Apa efficiently compared with non-targeted HMSNs nanoparticles in the acidic microenvironment of the tumor. *In vitro* studies showed that HACA nanoparticles exhibited the most potent cytotoxicity in osteosarcoma cells (143B cells) and significantly reduced cell proliferation, migration and invasion. Therefore, the drug-efficient release of antitumor effect of HACA nanoparticles is a promising way to treat osteosarcoma.

## 1 Introduction

Osteosarcoma is the most common primary malignant bone tumor, often occurring in adolescents, with a high tendency of local infiltration and distant metastasis, high recurrence rate and low survival rate (Jaffe, 2010; Freyer and Seibel, 2015; Spraker-Perlman et al., 2019). With the development of neoadjuvant chemotherapy and surgical techniques, amputation for osteosarcoma has been replaced by limb-preserving surgery and radiotherapy. The 5-year survival rate for patients has increased to about 70% (Ando et al., 2013). Unfortunately, the cure rate for osteosarcoma has not improved in the last 30 years (Liu et al., 2017). Therefore, there is an urgent need to find new treatment methods and drugs. Novel targeted therapies for osteosarcoma are currently attracting increasing interest. Apa is a novel, highly selective inhibitor of the vascular endothelial growth factor receptor 2 (VEGFR2) complex kinase that blocks downstream signaling of VEGFR2 and exerts antitumor effects in a variety of tumors (Hicklin and Ellis, 2005). Recent studies have shown that Apa has a growth inhibitory effect on osteosarcoma cells (Liu et al., 2017). For example, Han et al. reported that Apatinib inhibits cell proliferation and migration of osteosarcoma *via* activating LINC00261/miR-620/PTEN axis (Han et al., 2021). In addition, Li et al. also revealed that overcoming therapeutic failure in osteosarcoma *via* Apatinib-encapsulated hydrophobic poly(ester amide) nanoparticles (Li et al., 2020). However, Apa, like most chemotherapy drugs, cannot target tumor cells and has multi-organ toxicity, such as cardiotoxicity, hepatotoxicity and other systemic multi-organ toxicities (Maluccio and Covey, 2012; Gan et al., 2018). Nanoparticle-mediated targeted drug delivery systems are currently considered to be an effective strategy to address this problem. In addition, research on multifunctional nanoparticles as drug delivery systems continues to increase and is expected to be a new avenue for the treatment of tumors.

In recent years, HMSNs nanoparticles have received increasing attention due to their superior water dispersibility, biocompatibility and diverse biomedical applications (Ambrogio et al., 2011; Porta et al., 2013; Shen et al., 2013; Teng et al., 2013). As a drug delivery system, HMSNs nanoparticles have many advantages such as large specific surface area, uniform mesopore distribution, relatively large pore size, easy surface modification, good biocompatibility and high drug loading efficiency (Baù et al., 2009; Gao et al., 2011; Li et al., 2019). HMSNs are synthesized in various ways, such as Kirkendall effect method (Chen et al., 2010), soft/hard template method (Zhou et al., 2014), point-couple substitution method (Skrabalak et al., 2008), and hydrothermal method (Skrabalak et al., 2008). Most HMSNs are manufactured by the soft/hard template method, which requires complex steps to remove the template after the reaction. For example, in order to create internal void spaces, it is inevitable that the template is calcined at high temperatures or solvent extracted and cleaned with strong acids or bases (Skrabalak et al., 2008). This template-assisted method is effective for producing HMSNs with relatively narrow size distributions, but removing the core template may lead to agglomeration, or mutual adhesion to form larger particles (Lin et al., 2009). In addition, these techniques usually make it difficult to prepare spheres with sizes smaller than 100 nm. This limits their effective application as nanocarriers in the biological field, as they are extremely poorly absorbed by cells during drug delivery *in vivo* and tend to accumulate in the body (Lin et al., 2009). Highly dispersed mesoporous silica spheres with intact hollow interiors and through pores on the shell were fabricated (She et al., 2015). Cui et al. reported that the TBC/HMSN/P28 scaffold can promote proliferation and osteogenic differentiation of MC3T3-E1 cells *in vitro* and new bone tissue generation *in vivo*. HMSNs with small particle size were easier to enter bone tissue (Cui et al., 2018). Moreover, the lack of tumor specificity of HMSNs, like most antitumor drugs, also severely limits the application of HMSNs nanoparticles.

HMSNs can contain drugs through a simple physical adsorption process and release them independently. More importantly, the rational design of supramolecular nano-valve can prevent the early release of drugs during blood circulation and mitigate the non-selective damage to normal organs. Supramolecular nanovalves typically respond to biological signals (e.g., pH, enzymes) through the relative motion of large rings on a functionalized linear stem (Barat et al., 2015; Qiu et al., 2015). Based on the structural characteristics of β-cyclodextrin (β-CD) and the formation of “nanovalves” with various molecules (benzimidazole, etc.), its dissociation is closely related to the environmental conditions (temperature and pH) (Ma and Zhao, 2015). For instance, the acidic nature of the tumor microenvironment (TME) (Gong et al., 2018; Barve et al., 2020) allows the dissociation and disintegration of CD-based “nanovalves”. Meng et al. reported that CD nanovalves were modified on the surface of mesoporous silica spheres to achieve responsive release of the anticancer drug DOX, however, the loading of DOX by the mesoporous silica spheres was only about 2% and lacked tumor targeting ability (Meng et al., 2010). In addition, bisphosphonates have a relatively high binding affinity for hydroxyapatite compared to other calcium minerals such as calcium oxalate, calcium carbonate or calcium pyrophosphate (Zaheer et al., 2001; Lenkinski et al., 2003; Torres Martin de Rosales et al., 2009; Hyun et al., 2014). This advantage has led to the widespread use of bisphosphonates in bone targeting. Take ALN as an example, it can be used not only as a bone disease drug, but also as a bone targeting ligand (Sun et al., 2016). Guven et al. used bisphosphonic acid groups as biomineralization-inducing sites, recruiting positive ions [mainly calcium ions (Ca^2+^)] and negative ions [mainly phosphate groups (PO_4_^3-^)]. Nanoparticles reach the tumor tissue and form a mineral barrier around it, effectively inhibiting the proliferation and migration of cancer cells (Guven et al., 2018). In addition, as a diphosphonate, ALN attenuates the bone destruction involved in osteosarcoma by inhibiting the activity of osteoclasts, which also contributes to delaying the possibility of pulmonary metastasis (Jiang et al., 2022).

Therefore, a supramolecular “nanovalve” - β-CD-benzimidazole (BM) - anchored on the outer surface of small-sized HMSNs was designed and loaded with Apa, then modified with bone-targeting ALN. The nanoparticles can act as a bifunctional nanosystem, which not only targets bone tissue, but also effectively releases the antitumor drug Apa in the low pH microenvironment, reducing its toxic effects on the organism. Thus, the design of this nanoparticle provides a new paradigm for the treatment of bone tumors.

## 2 Materials and methods

### 2.1 Materials

Hexadecyltrimethylammonium bromide (CTAB) was purchased from sigma (Shanghai, China); Octanol, cyclohexane, X-100 Trition, ammonia, anhydrous ethanol, acetic acid, tetraethylorthosilicate (TEOS), (3-Aminopropyltriethoxysilane (APTES), acetone, 2-benzimidazolepropionic acid (BM) were purchased from Aladdin Ltd. (Shanghai, China). NHS-PEG-COOH (PEG) was purchased from Tuo Yang Biotechnology (Shanghai, China). Alendronate sodium trihydrate (ALN), β-Cyclodextrin (CD), N-Hydroxysuccinimide-1-hydroxypyrrolidine-2,5-dione (NHS), (1- (3-Dimethylaminopropyl)-3-ethylcarbodiimide hydrochloride (EDC·HCl), Dimethyl sulfoxide (DMSO) were purchased from Beyoytime (Shanghai, China); 3- (4,5-dimethyl-2-thiazolyl)-2,5-diphenyl-2-H-tetrazolium bromide (MTT), Crystalline violet staining solution, Chlorin e6 (Ce6), 4% Paraformaldehyde, 4,6-Diamidino-2-phenylindole dihydrochloride (DAPI), Pore polycarbonate film (pore size 8 mm), Matrigel were purchased from Solarbio (Beijing, China). All chemicals were used without further purification. Distilled and deionized water was used throughout the experiments.

### 2.2 Methods

#### 2.2.1 Preparation of HACA

##### 2.2.1.1 Preparation of HMSNs

Cyclohexane 145.2 mL, n-octanol 35.2 mL, CTAB (30 mg, 0.08 mmol) and X-100 Trition 35.76 g were mixed thoroughly in a round bottom flask and 8.8 mL of distilled water containing 20 ul of APTEs was added separately. After thorough stirring, NH_3_·H_2_O (1.6 mL) and TEOS (1.6 mL) were added respectively. After stirring for 24 h, the reaction was terminated by adding an appropriate amount of acetone until a flocculent substance appeared. After centrifugation, the white precipitate was obtained, and the appropriate amount of distilled water and anhydrous ethanol were added and stirred thoroughly until completely dissolved and then centrifuged, and each of such steps was operated three times. The resulting white precipitate was put into a round bottom flask with an appropriate amount of acetic acid and stirred for 4 h, then centrifuged, washed again with distilled water for 3 times, and finally dried at 60°C to obtain HMSNs for further use.

##### 2.2.1.2 Preparation of HMSNs/BM and HMSNs/BM-Apa (HA)

BM (100 mg, 0.53 mmol), EDC (201.58 mg, 1.05 mmol) and NHS (121.01 mg, 1.05 mmol) were added to deionized water (30 mL) in turn and stirred thoroughly for 30 min, followed by the addition of the above prepared HMSNs (50 mg) and Apa (50 mg, 0.10 mmol), dissolve completely after sonication, continue to stir for 24 h and then centrifuge and dry at 60°C.

##### 2.2.1.3 Preparation of OTs-β-CD (CD)

First, cyclodextrin (42 g, 0.037 mol) was dissolved in 300 mL distilled water. NAOH (0.333 g/mL, 12 mL) solution was added to make the solution turn yellow. Then p-toluenesulfonyl chloride (6.06 g, 0.032 mol) in 20 mL acetonitrilewas added into the above solution within 10 min. White precipitate appeared immediately. Afterwords, the solution was stirred for 2.5 h, filtered, neutralized with hydrochloric acid until slightly alkaline. After filtration, the product was recrystallized twice and dried to obtain OTs-β-CD. OTs-β-CD (1.5 g, 1.17 mmol) is added to 5 mL of ethylenediamine under nitrogen atmosphere and stirred for 24 h at 60 °C. Finally, the product is filtered in a large amount of ethanol to obtain white precipitate and dried for further use.

##### 2.2.1.4 Preparation of CD-PEG-ALN (CA) and HACA

Firstly, CD (58.85 mg, 0.05 mmol) and PEG (100 mg, 0.05 mmol) were added to deionized water (20 mL) and stirred for 24 h. Then EDC (19.17 mg, 0.10 mmol), NHS (11.51 mg, 0.10 mmol) and ALN (27.11 mg, 0.10 mmol), stirred again for 24 h and then dialyzed overnight, and finally lyophilized to obtain CD-PEG-ALN (CA). Dissolving CD (5 mg), CD-PEG (5 mg) and CA (5 mg) in a certain amount of deuterium water (500 ul) and being analyzed by Nuclear magnetic resonance spectrometer (AVANCE 600, Germany). CD-PEG: ^1^H NMR (400 MHz, D_2_O) δ 4.88 (s, 1H), 3.72 (dt, *J* = 15.6, 11.0 Hz, 4H), 3.52 (s, 18H), 3.05 – 3.02 (m, 1H), 2.77 (s, 1H), 2.50 (s, 1H), 2.26 (s, 1H). CD-PEG-ALN: 1H NMR (400 MHz, d2o) δ 4.88 (d, *J* = 3.8 Hz, 2H), 3.80 – 3.61 (m, 5H), 3.52 (s, 31H), 3.07 – 2.82 (m, 5H), 2.69 (s, 2H), 2.56 (s, 1H), 1.82 (s, 2H), 0.89 (t, *J* = 7.2 Hz, 1H). Finally, CA (20 mg) was mixed with HA (20 mg) for 24 h and centrifugally dried to obtain the final product HACA. Similarly, in the preparation of HMSNs/BM-CD-PEG-ALN (HCA) and HMSNs/BM-Apa-CD-PEG (HAC), Apa and ALN are not added in the above preparation process, and the rest of the steps are obtained in the same way.

### 2.3 Characterization

The morphology of the synthesized HMSNs and HACA nanoparticles was observed by Tecnai 12 transmission electron microscopy (TEM, Philips Netherlands). The TEM samples were sonicated and dispersed in ethanol on a deposited porous carbon grid. Fourier transform infrared (FTIR, Nicolet 6700, Thermo Fisher, United States) spectroscopy is used to analyze changes in the biochemical composition of a sample. Following the KBr pellet method, a small quantity of the samples was grinded with KBr powder. All measurements were performed with transmittance mode and recorded in the spectral range of 4000–400 cm^−1^ for 16 scans and with a spectral resolution of 4 cm^−1^. The UV–Vis absorption of the sample and quantitative analy-sis was performed by an UV–vis spectrophotometer (Nicolet Evolution 300, Thermo Fisher, United States). The samples were evenly dispersed in DMSO and the UV–Vis absorption spectra of the samples were measured at room temperature. The data was qualitatively and quantitatively analyzed by the standard curve method.

### 2.4 Cell culture

Osteosarcoma cell line 143B was purchased from Jihe Biotechnology (Shanghai, China). This cell line was cultured with DMEM medium containing 10% fetal bovine serum (FBS) and 1% penicillin/streptomycin. The culture was maintained at 37 °C and under humidified 5% CO_2_. These cells were passaged every 2 days. Once the fusion state was reached, the cells were collected by trypsin (containing 0.25% EDTA) digestion for further experiments.

### 2.5 *In vitro* targeted uptake of nanoparticles

The affinity of ALN for bone minerals was investigated for non-targeted HA, HAC, compared with targeted HACA nanoparticles. The same masses of HA, HAC and HACA nanoparticles were taken with 5 times the mass of hydroxyapatite and centrifuged for 24 h, respectively. The precipitates were washed several times with appropriate amount of deionized water and dried in an oven for scanning electron microscopy (SEM, SIGMA 300, Germany) characterization.

### 2.6 Intracellular nanoparticle uptake

The intracellular behavior of HACA nanoparticles containing fluorescent Ce6 was observed by laser scanning confocal microscopy (LSCM, Leica, TCS SP5, Germany). 143B cells were inoculated in a 6-well plate with a sterile coverslip at the bottom of each well, and 1.5 × 10^5^ cells were inoculated per well. Cells were incubated with DMEM medium containing 10% fetal bovine serum (FBS) and 1% penicillin/streptomycin in a humidified 5% CO_2_ incubator at 37 °C. 200ug/mL of HMSNs/BM-Ce6-Apa-CD-PEG-ALN (HACA-Ce6) nanoparticles were added to the culture medium. After 24 h of incubation, the medium was removed and the cells were rinsed 3 times with PBS. Cells were fixed in 4% paraformaldehyde in PBS for 20 min and then gently rinsed with PBS 3 times. The fixed cells were stained with DAPI for 15 min, washed 3 times with PBS and the coverslips were fixed on glass microscope slides. Finally, the samples were observed under LSCM.

### 2.7 Loading and release of Apa *in vitro*

First, a certain amount of Apa was dissolved in DMSO, and mixed with an equal mass of HMSNs/BM in deionized water. The suspension was centrifuged at 10,000 r min^-1^ for 15 min at 4°C and dried to obtain HA, and the supernatant was used for further quantitative analysis. Apa was quantified by photometer absorption spectroscopy using the standard curve method. The standard curve was established by measuring the highest absorption peak at 260 nm for different concentrations of Apa. The absorbance was measured by ultramicro spectrophotometer and the fitted standard curve was: Y = 38.904X-0.2601, *R* ^2^ = 0.996. Therefore, the drug loading capacity (LC) of Apa and the Apa loading efficiency (EE) are calculated by the following equation:

$$LC\%=\frac{Amount\;of\;loaded\;Apa}{Total\;weight\;of\;nanoparticles}\;*100\%$$

$$EE\%=\frac{Amount\;of\;loaded\;Apa}{Amount\;of\;feeding\;Apatinib}\;*100\%$$

The prepared HACA solutions were transferred to dialysis bags, sealed, and immersed in PBS buffer at pH 6.0 and pH 7.4, respectively. The dialysis experiment was maintained at 37 °C with a continuous vibration of 100 rpm. The sample solutions were extracted at 0.25, 0.5, 1, 2, 4, 6, 8, 10, 12, 24, 24, 48, 72, 96 and 120 h, respectively. Replenish with equal amounts of fresh PBS. The release of Apa was quantified by standard curve method. The release profile of Apa was investigated by calculating the cumulative release rate of Apa.

### 2.8 Cell biocompatibility of HCA and cell viability assay of HACA

The cytotoxicity of HCA and HACA were assessed using MTT colorimetric assay. 143B cells were inoculated into 96-well plates at a density of 5×10^3^ cells/well and incubated at 37 °C in a 5% CO_2_ incubator for 24 h. Then, DMEM medium containing 10% FBS was configured with different concentrations of HCA and HACA (0, 50, 100, 200, 400, 600, 800, 1600, 2000 ug/mL). After 24 h, 10 ul of MTT solution was added to each well and the cells were then incubated in the dark for 4 h. Afterwards, the supernatant was aspirated and washed 3 times with PBS, DMSO (200 ul) was quickly added to each well, and then placed on a shaker for 10 min. The absorbance values were then measured at 570 nm using a multifunctional enzyme marker (TECAN Austria). The following equation is used to calculate cell viability: Cell viability (%) = (OD of experimental group - OD of blank group)/(OD of control group - OD of blank group)*100% (Du et al., 2017). Each sample was tested in triplicate.

### 2.9 Cell migration assay, *in vitro* transwell cell invasion assay and cell clone formation assay

Scratching experiments were performed to investigate the effect of HACA nanoparticles on the migration ability of 143B cells. The experimental groups included HMSNs, Apa, HAC, HACA and control group with the pH of the medium was set to 6.0. The 143B cells were inoculated in 6-well plates with 5 × 10^5^ cells per well. After 24 h of incubation, the cells form a monolayer and cover approximately 80% of the bottom of the well. The cell monolayer was scraped using the pipette tip to create a scratch, and then carefully rinsed 3 times with PBS. HMSNs, Apa, HAC, and HACA nanoparticles were added, respectively, and then incubated for 24 h. The *in vitro* wound formation and its repopulation were assessed by inverted microscopy.

The *in vitro* invasive capacity of 143B cells was determined by a modified Boyden chamber system. The 24-Transwell Boyden Chamber unit (Neuroprobe, United States) is divided into two compartments with a porous polycarbonate membrane (8 mm pore size) between the upper and lower chambers. Matrigel is used to simulate the barrier of the extracellular matrix, and its coating on the membrane forms a thin layer of barrier. 143B cell suspension (2.0 × 10^5^ cells/well, DMEM medium) was inoculated onto the gel membrane of the top chamber. The transwell was incubated for 24 h, and then the non-invasive cells were removed with a cotton swab. Migrated cells were fixed with 4% paraformaldehyde for 30 min and stained with crystal violet solution (0.1%) for 15 min. Wash the Transwell insert several times. Observe and count the invading cells under an inverted microscope using white light.

For cell colony formation assay, 143B cell suspension (500 cells/well, DMEM medium) was inoculated onto 6-well plates. After 24 h, HMSNs, Apa, HAC, and HACA materialswere added, respectively. After 5 days of incubation, the supernatant was aspirated, fixed with 4% paraformaldehyde for 15 min, and stained with crystal violet solution (0.1%) for 15 min. The proliferating cells were observed and counted under an inverted microscope using the same white light.

### 2.10 Statistical analysis

All data were expressed as mean ± standard deviation (Mean ± SD), and statistical analyses were performed using SPSS 19.0 software and plotted using GraphPad Prism8 software. Student’s t-test was used to compare statistical differences between the two groups and One-way ANOVA was used to test for differences between multiple groups. P< 0.05 was considered statistically different.

## 3 Results and discussion

### 3.1 Morphological characterization and analysis

**SCHEME 1:**
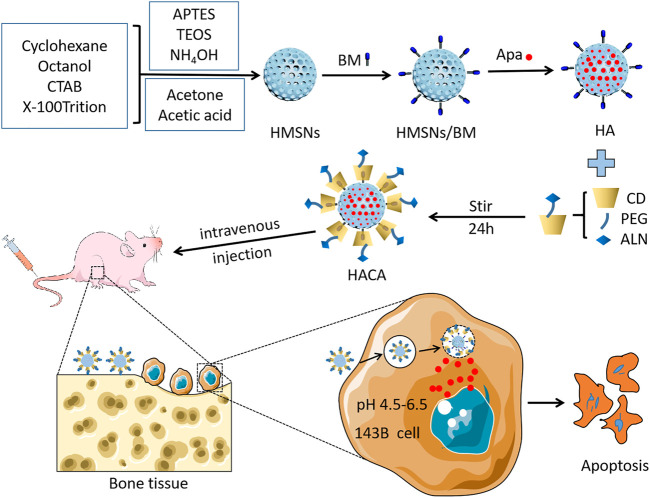
Schematic illustration of the fabrication of the HACA and their application for pH-responsive drug release after specific binding with bone tissue.

Scheme 1 showed the detailed preparation of HACA. HMSNs were synthesized by the one-step method using a modified water-in-oil (W/O) reverse microemulsion system containing APTES, X-100 Triton, n-hexanol, cyclohexane and water, a well-studied system for the synthesis of silica nanoparticles with the size less than hundred nanometers. After the addition of TEOS, the polymerization was triggered with NH_4_OH and continued for 24 h. Afterwards, acetone was added to terminate the reaction. The HMSNs underwent a spontaneous morphology change from solid to hollow when they were washed with ethanol and water by such a simple etching-free strategy. Finally, the CTAB template was removed by acidic ethanol extraction to obtain homogeneous HMSNs. Then, BM is affixed to the amine group on the surface of HMSNs by forming an “amide bond” and HMSNs/BM is formed. Apa, used as a model anticancer drug, was loaded into the porous structure of the HMSNs/BM shell.

**FIGURE 1:**
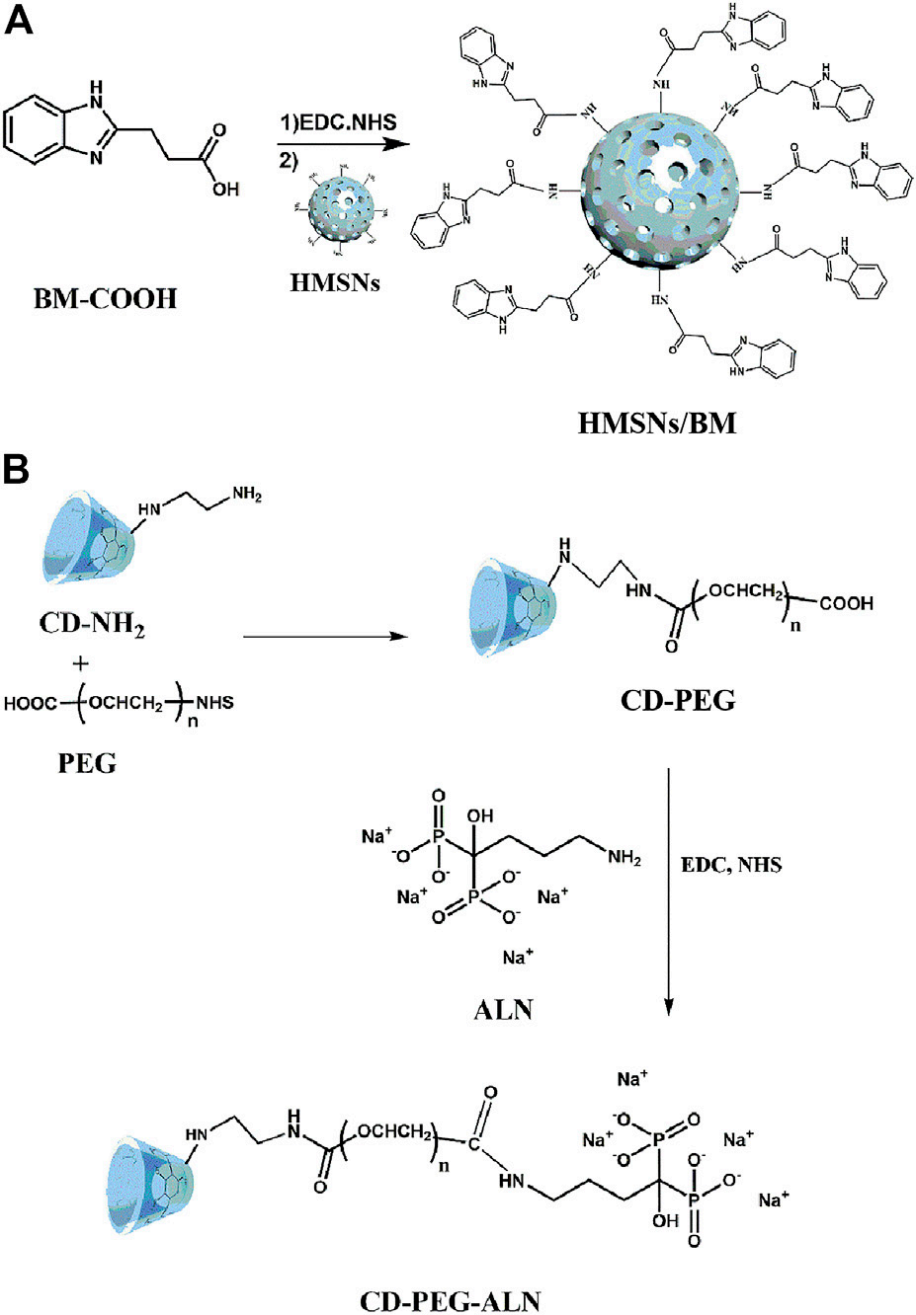
The synthesis of HMSNS/BM **(A)** and CD-PEG-ALN **(B)**.

ALN-modified CD with bone-targeting ability binds to a linear stem provided by the HA surface to obtain HACA (Figure 1). In addition, CD-PEG and CD-PEG-ALN (CA) were characterized by ^1^H nuclear magnetic resonance (NMR) spectroscopy in the supporting information. The peaks at approximately 3.51 ppm were assigned to the repeating unit -O-CH_2_-CH_2_- on the PEG chain (Supplementary Figure S1) and the characteristic peaks at 2.68 ppm were assigned to the proton in -NH-CO- (Supplementary Figure S2), which demonstrated that the successful synthesis of CD-PEG and CD-PEG-ALN.

**FIGURE 2:**
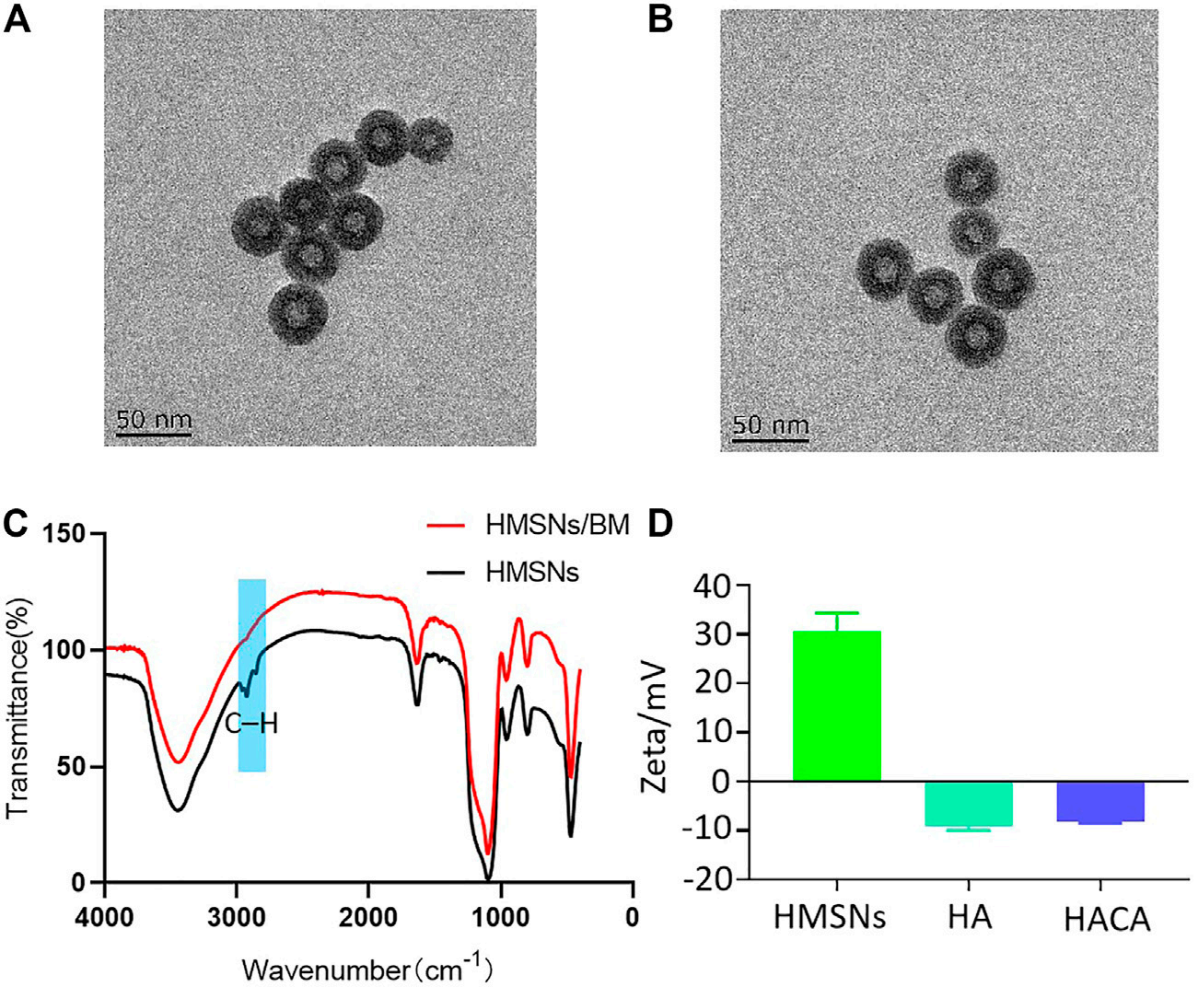
Preparation and characterization of HACA nanoparticles. **(A**, **B)** Transmission electron microscopy images of HMSNs and HACA, respectively. **(C)** FTIR spectroscopy of HMSNs and HMSNs/BM. **(D)** Zeta potential of HMSNs, HA and HACA. Scale bar is 50 nm.

The morphology and structure of HMSNs and HACA nanoparticles were observed by transmission electron microscopy. As shown in Figures 2A, B, the HMSNs and HACA nanoparticles are almost spherical with uniform size distribution and the range of diameters is about 30–40 nm, while the morphological and structural changes are not obvious. The FTIR spectrum for HMSNs (Figure 2C) showed the absorption band at 2920 and 2848 cm^–1^, belonging to C–H stretching vibrations, experienced a substantial reduction in HMSNs/BM. The two curves of the remaining parts approach each other, indicating that HMSNs-BM basically retains the structural characteristics of HMSNs. These data further demonstrate the presence of BM on the surface of the prepared HMSNs. In addition, the zeta potential analysis (Figure 2D) showed that the average potential of the positively charged HMSNs was 26 mV, while the average potential of the synthesized HA was −9 mV. Similarly, the potential of HACA was −7 mV which became smaller, further indicating the successful synthesis of ALN on the surface of HACA nanoparticles.

### 3.2 Cellular uptake of HACA *in vitro*

**FIGURE 3:**
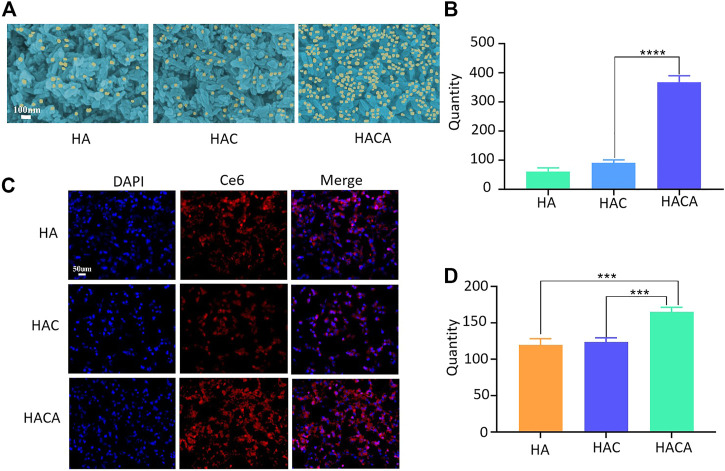
Schematic diagram of *in vitro* cellular uptake of nanoparticles. **(A)** Scanning electron microscopy images of HA, HAC and HACA bound to hydroxyapatite. **(B)** Analysis of nanoparticles numbers bound to hydroxyapatite. **(C)** Intracellular uptake of HA, HAC and HACA in 143B cells. Confocal laser scanning microscopy images of 143B cells depict intracellular uptake of Ce6-labeled HA, HAC and HACA (red), where the nuclei are stained with DAPI (blue). **(D)** Analysis of the number of 143B cells engulfed by Ce6-labeled nanoparticles. *p < 0.05, **p < 0.01 and ***p < 0.001 vs. blank. Scale bars are 100 μm.

The targeting of ALN to bone tissue is an important prerequisite to ensure the effectiveness of HACA nanoparticles for clinical application. Hydroxyapatite (HAP) is the most abundant mineral component in bone tissue, which was chosen to simulate the bone microenvironment for *in vitro* experimental validation. *In vitro* targeting ability of HACA were simulated through HAP experiments. As shown in Figures 3A, B, the same concentrations of HA, HAC, HACA were co-incubated with HAP for 24 h. After scanning by electron microscopy, it was found that the HAP surface.

Was covered by a large amount of HACA. However, HA and HAC nanoparticles without ALN modification were observed with a smaller number of nanoparticles on the surface of HAP.

In addition, in order to investigate the uptake of nanoparticles by cells *in vitro*, Ce6 was operated in parallel with Apa during the synthesis process. CLSM was used to visualize the internalization and intracellular distribution of HA-Ce6、HAC-Ce6 and HACA-Ce6 in 143B cells following incubation for 24 h. As shown in Figures 3C, D, the red fluorescence strengthened with the different nanoparticles and the targeted HACA-Ce6 particles were more likely to enter tumor cells compared with non-targeted HA-Ce6 and HAC-Ce6 nanoparticles. These results suggest that ALN has a good bone targeting ability, which can make the nanoparticles target bone tissue more and enter tumor cells more effectively.

### 3.3 Loading and releasing of Apa *in vitro*

**FIGURE 4:**
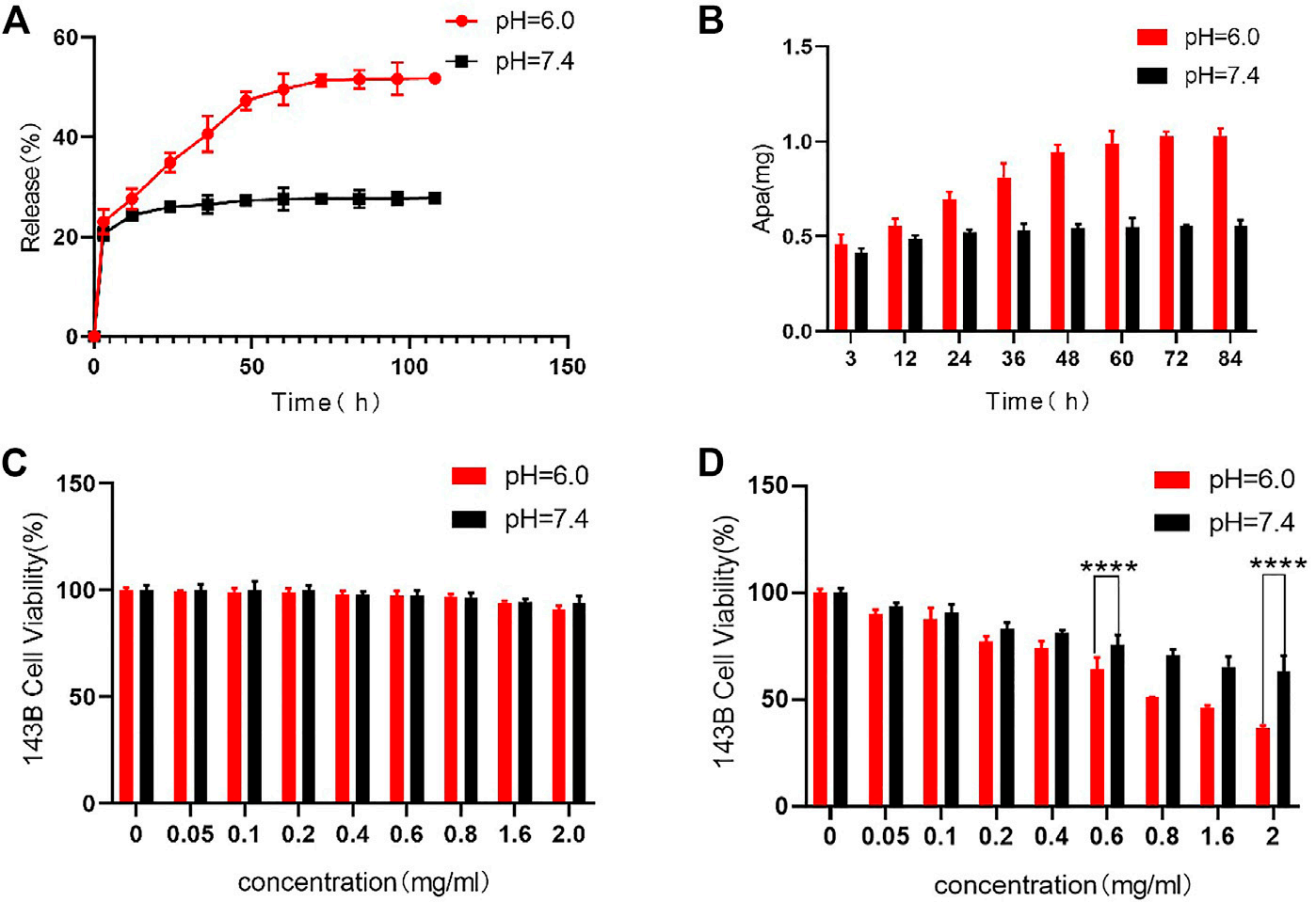
*In vitro* release of Apa and *in vitro* cytotoxicity to 143B cells. **(A) (B)** Release curves of Apa from HACA under different pH conditions. **(C)** Effect of HAC on 143B cell viability at different pH. **(D)** Effect of different concentrations of HACA on 143B cell viability under different pH conditions. *p < 0.05, **p < 0.01 and ***p < 0.001 vs. blank.

As an anti-tumor drug, Apa has strong tumor treatment ability, but its non-specific effect brings toxic side effects to the normal tissues, which limits the clinical application. HACA nanoparticles are mainly composed of 3 parts, including HMSNs/BM, Apa, and CD-PEG-ALN. HACA nanoparticles have high drug LC and drug EE of 65% and 25%, respectively. The ideal drug carrier should not only have a good drug loading rate, but also have environmentally controlled release capability. The drug release curves of Apa in Figures 4A, B were conducted by dispersing HACA in different PBS buffers (pH 6.0 and 7.4, respectively). The Apa in HACA nanoparticles was released more easily at pH 6.0. Almost 58% of Apa was simultaneously released from the particles. However, only 24% of the Apa in HACA was released after 48 h of immersion in the pH 7.4 environment. This is mostly attributed to the “nano-valve” made of CD disintegrating in an acidic environment, which exposes the pore channels on HMSNs and causes the release of Apa.

### 3.4 Cell biocompatibility and the evaluation of anti-tumor effect of HACA *in vitro*

HCA and HACA nanoparticles were incubated with 143B cells to assess their biocompatibility and tumor treatment effect. As shown in Figure 4C, cell viability measured by the MTT assay after being incubated with HCA nanoparticles for 48 h in different pH environments. When the concentration of HCA nanoparticles increased to 2 mg/mL, the cell survival rate remained above 95%. This result indicates that HCA nanoparticles have no significant toxicity on the cellular activity and can be used as ideal drug carriers for tumor treatment. While as shown in Figure 4D, when HACA nanoparticles were co-incubated with 143B cells in medium of different pH, the survival rate of 143B cells gradually decreased as the concentration of HACA nanoparticles increased. In comparison with the same concentration of HACA nanoparticles co-incubated with 143B in different pH conditions, the cell survival rate.

Was significantly lower in pH 6.0 than in pH 7.4. Since the tumor environment and other areas of inflammation tend to be more acidic than the normal physiological environment, it is expected that these areas of higher acidity would permit greater Apa release. The pH-responsive release of Apa can be utilized to achieve efficient intracellular delivery.

### 3.5 Effect of HACA on migration, invasion and proliferation abilities

**FIGURE 5:**
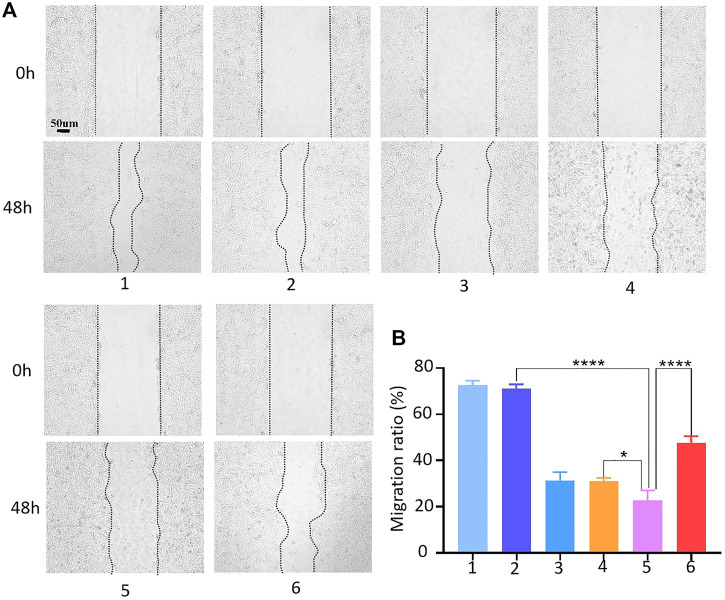
Cell migration assay. **(A)** Conditioned medium-mediated cell migration assay of 143B cells at 0–48 h. **(B)** Mobility % = (S_0_-S_48_)/S_0_*100%, where S_0_ and S_48_ indicate the area of the scratch at 0h and 48h. 1, 2, 3, 4, 5 and 6 represent blank control, HMSNs, Apa, HA, HACA in medium pH = 6.0 and HACA in medium pH = 7.4, respectively. *p < 0.05, **p < 0.01 and ***p < 0.001 vs. blank. The scale bar is 100 μm.

The effects of HACA nanoparticles on migration, invasion and proliferation ability of 143B cells were also assessed. The process of scratch healing was observed with an optical microscope and the cell migration rate was calculated by ImageJ software. As shown in Figures 5A, B, compaired with the strongest migration ability in control group, HMSNs, free Apa, HA and HACA significantly inhibited the migration of 143B cells. Notably, HACA was much more effective in retarding the migration of 143B cells at pH 6.0 than that of in neutral condition. This is mainly due to that HACA nanoparticles released more Apa in the acidic tumor environment. These results showed that the HACA nanoparticles could reduce the migration ability of 143B cells, which will effectively prevent the metastasis of tumor cells.

**FIGURE 6:**
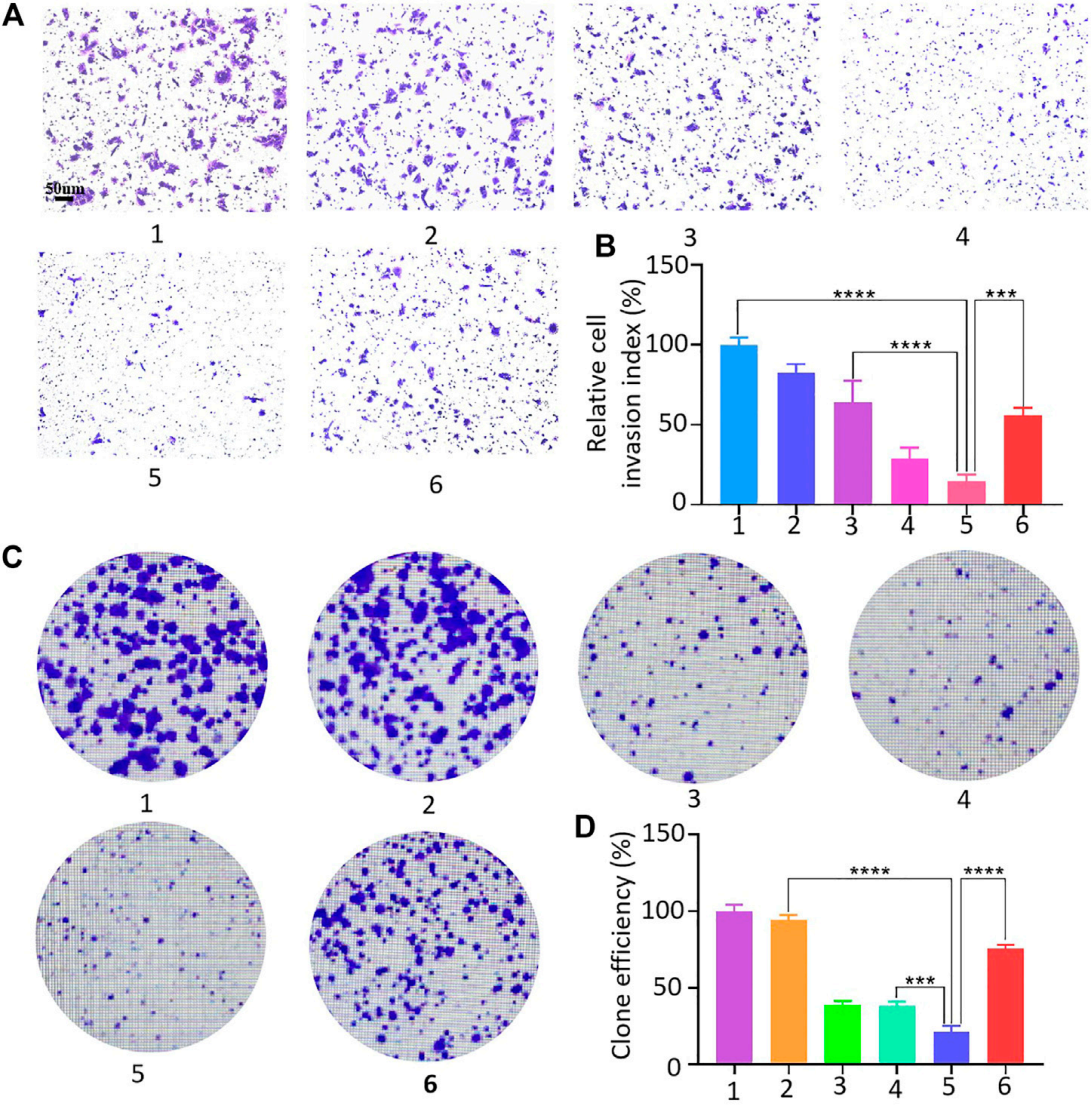
143B cell invasion assay and clone formation assay. **(A**–**D)** 143B cell invasion and proliferation capacity assessment: 1, 2, 3, 4, 5 and 6 represent control, HMSNs, Apa, HA, HACA in medium pH = 6.0 and HACA in medium pH = 7.4, respectively. *p < 0.05, **p < 0.01 and ***p < 0.001 vs. blank. Scale bars are 100 μm.

The 143B cell suspension was spread in Transwell chambers, and HMSNs, Apa, HA, HAC, and HACA nanoparticles were added into the chamber, and PBS solution was added as the control group. The number of cells that degrade the basement membrane and pass through the polycarbonate membrane was observed under a microscope. Figures 6A, B showed that 143B cells in the control group had strong invasion ability and most cells can pass through the polycarbonate membrane after 48 h. While the invasion ability of 143B cells in the experimental groups were weakened significantly, and the 143B cells in the HACA nanoparticles group at pH 6.0 condition could barely pass through the polycarbonate membrane, so the cell invasion ability was greatly reduced.

The effect of HACA nanoparticles on the proliferative capacity of 143B cells were investigated. As shown in Figures 6C, D, similarly, free Apa, HA and HACA significantly inhibited the proliferative capacity of 143B cells, and HACA was much more effective in retarding the proliferative capacity of 143B cells. When the pH was at 6.0, the HACA nanoparticle group showed the strongest inhibition of 143B cell proliferation and significantly reduced the colony formation. These results demonstrated that HACA nanoparticles could significantly inhibit the proliferation of 143B cells.

## 4 Discussion and conclusion

In this report, nanoplatforms HACA was successfully constructed basing on the small-sized HMSNs as the template and CD-BM supramolecular assemblies as the valves, which can load and control the release of Apa and target bone tissue. This method is easy and economical for large-scale preparation. The HACA nanoparticles were in the size of about 30–40 nm. When incubated with different concentrations of HCA nanoparticles within 143B cells, and the cell vitality is not reduced significantly which showed the good biocompatibility of the nanocarriers. A standard curve was established according to the highest absorption peak at 260 nm of Apa solutions with different concentrations, and it was obtained that the nanoparticles had a high drug loading efficiency, up to 25%. ALN-modified nanoparticles are easier to bind to hydroxylapatite and have good bone tumor targeting capabilities. After co-incubation of HACA-Ce6 with 143B cells, the fluorescence was observed under CLSM, and the results showed that HACA-Ce6 nanoparticles were more easily absorbed by 143B cells. The pH of tumor tissues is lower than the normal physiological environment due to the high metabolism of tumor cells, which can lead to lactic acid accumulation. Therefore, the release of Apa in HACA was examined by simulating the tumor microenvironment and normal physiological environment *in vitro.* The results showed that HACA nanoparticles released more Apa at pH = 6.0. After 48 h, the release rate of Apa from HACA was about 58%, while in normal physiological environment, the release rate of Apa from HACA was only 25%. Furthermore, tumors have the characteristics of unlimited proliferation, local invasion, and distant metastasis. Cell motility plays a crucial role in many physiological and pathological processes, including embryonic development, wound healing, and metastasis of tumor cells. To study the effect of tumor cell migration ability on tumor metastasis and recurrence, cell scratch assay is considered as a simple, effective and direct method to observe cell migration process *in vitro.* Cells at the edge of the scratch will gradually enter the blank area, allowing the “scratch” to heal, simulating the process of cell migration *in vivo* (Liang et al., 2007). *In vitro* cell invasion ability is one of the distinguishing features of tumor cell metastasis. The Transwell cell invasion assay simulates tumor cell invasion and assesses the invasive ability of tumor cells *in vitro*. The assessment of HACA nanoparticles on 143B cell migration, invasion and proliferation were performed by simulating the tumor microenvironment *in vitro*. The results showed that the HACA nanoparticles exhibited a strong ability to inhibit the proliferation, invasion, and migration of 143B cells when the pH of the microenvironment is 6.0.

In summary, the HACA nano-drug delivery system was successfully prepared and used for the treatment of osteosarcoma *in vitro*. HACA nanoparticles were spherical with an average diameter of about 30–40 nm and they have a high drug loading efficiency (25%) and pH-responsive slow-release capacity. It was shown that HCA nanoparticles had low cytotoxicity to cells, but HACA could effectively inhibit the proliferation, migration and invasion ability of 143B cells. It provides new ideas and methods for clinical oncology treatment. Although HACA nanoparticles have certain advantages and application prospects for the treatment of osteosarcoma, they also have shortcomings. For example, 1) the experiments lacks *in vivo* validation, and the complexity of the tumor microenvironment *in vivo* is much higher than that simulated *in vitro.* 2) The toxicity of HACA nanoparticles to human normal cells lacks verification. Whether it will cause toxic side effects to normal tissues and organs is unknown.

## Data Availability

The original contributions presented in the study are included in the article/Supplementary Material, further inquiries can be directed to the corresponding authors.

## References

[B1] Ambrogio M. W. Thomas C. R. Zhao Y. L. Zink J. I. Stoddart J. F. (2011). Mechanized silica nanoparticles: A new frontier in theranostic nanomedicine. Acc. Chem. Res. 44 (10), 903–913. 10.1021/ar200018x 21675720 PMC3196789

[B2] Ando K. Heymann M. F. Stresing V. Mori K. Redini F. Heymann D. (2013). Current therapeutic strategies and novel approaches in osteosarcoma. Cancers 5 (2), 591–616. 10.3390/cancers5020591 24216993 PMC3730336

[B3] Barat R. Legigan T. Tranoy-Opalinski I. Renoux B. Peraudeau E. Clarhaut J. (2015). A mechanically interlocked molecular system programmed for the delivery of an anticancer drug. Chem. Sci. 6 (4), 2608–2613. 10.1039/c5sc00648a 29308165 PMC5649224

[B4] Barve A. Jain A. Liu H. Zhao Z. Cheng K. (2020). Enzyme-responsive polymeric micelles of cabazitaxel for prostate cancer targeted therapy. Acta Biomater. 113, 501–511. 10.1016/j.actbio.2020.06.019 32562805 PMC7423752

[B5] Baù L. Bartova B. Arduini M. Mancin F. (2009). Surfactant-free synthesis of mesoporous and hollow silica nanoparticles with an inorganic template. Chem. Commun. (Camb) 2009, 7584–7586. 10.1039/b917561j 20024287

[B6] Chen Y. Chen H. Guo L. He Q. Chen F. Zhou J. (2010). Hollow/rattle-type mesoporous nanostructures by a structural difference-based selective etching strategy. ACS Nano 4 (1), 529–539. 10.1021/nn901398j 20041633

[B7] Cui W. Liu Q. Yang L. Wang K. Sun T. Ji Y. (2018). Sustained delivery of BMP-2-related peptide from the true bone ceramics/hollow mesoporous silica nanoparticles scaffold for bone tissue regeneration. ACS Biomater. Sci. Eng. 4 (1), 211–221. 10.1021/acsbiomaterials.7b00506 33418690

[B8] Du F. Lou J. Jiang R. Fang Z. Zhao X. Niu Y. (2017). Hyaluronic acid-functionalized bismuth oxide nanoparticles for computed tomography imaging-guided radiotherapy of tumor. Int. J. Nanomedicine 12, 5973–5992. 10.2147/ijn.s130455 28860761 PMC5573055

[B9] Freyer D. R. Seibel N. L. (2015). The clinical trials gap for adolescents and young adults with cancer: Recent progress and conceptual framework for continued research. Curr. Pediatr. Rep. 3 (2), 137–145. 10.1007/s40124-015-0075-y 30613438 PMC6319956

[B10] Gan L. Liu Z. Sun C. (2018). Obesity linking to hepatocellular carcinoma: A global view. Biochim. Biophys. Acta Rev. Cancer 1869 (2), 97–102. 10.1016/j.bbcan.2017.12.006 29366974

[B11] Gao Y. Chen Y. Ji X. He X. Yin Q. Zhang Z. (2011). Controlled intracellular release of doxorubicin in multidrug-resistant cancer cells by tuning the shell-pore sizes of mesoporous silica nanoparticles. ACS Nano 5 (12), 9788–9798. 10.1021/nn2033105 22070571

[B12] Gong F. Cheng L. Yang N. Jin Q. Tian L. Wang M. (2018). Bimetallic oxide MnMoO(X) nanorods for *in vivo* photoacoustic imaging of GSH and tumor-specific photothermal therapy. Nano Lett. 18 (9), 6037–6044. 10.1021/acs.nanolett.8b02933 30141945

[B13] Guven M. N. Altuncu M. S. Bal T. Oran D. C. Gulyuz U. Kizilel S. (2018). Bisphosphonic acid-functionalized cross-linkers to tailor hydrogel properties for biomedical applications. ACS Omega 3 (8), 8638–8647. 10.1021/acsomega.8b01103 31458994 PMC6644954

[B14] Han G. Guo Q. Ma N. Bi W. Xu M. Jia J. (2021). Apatinib inhibits cell proliferation and migration of osteosarcoma via activating LINC00261/miR-620/PTEN axis. Cell Cycle 20 (18), 1785–1798. 10.1080/15384101.2021.1949132 34424120 PMC8525947

[B15] Hicklin D. J. Ellis L. M. (2005). Role of the vascular endothelial growth factor pathway in tumor growth and angiogenesis. J. Clin. Oncol. 23 (5), 1011–1027. 10.1200/jco.2005.06.081 15585754

[B16] Hyun H. Wada H. Bao K. Gravier J. Yadav Y. Laramie M. (2014). Phosphonated near-infrared fluorophores for biomedical imaging of bone. Angew. Chem. Int. Ed. Engl. 53 (40), 10844–10848. 10.1002/ange.201404930 PMC4221277 25139079

[B17] Jaffe N. (2010). “Osteosarcoma: Review of the past,” in Impact on the future. The American experience. Pediatric and adolescent osteosarcoma (Boston, MA: Springer US), 239–262. 10.1007/978-1-4419-0284-9_12 20213394

[B18] Jiang Z. Liu Y. Shi R. Feng X. Xu W. Zhuang X. (2022). Versatile polymer-initiating biomineralization for tumor blockade therapy. Adv. Mater 34 (19), e2110094. 10.1002/adma.202110094 35202501

[B19] Lenkinski R. E. Ahmed M. Zaheer A. Frangioni J. V. Goldberg S. (2003). Near-infrared fluorescence imaging of microcalcification in an animal model of breast cancer. Acad. Radiol. 10 (10), 1159–1164. 10.1016/s1076-6332(03)00253-8 14587634

[B20] Li X. Wang L. Wang L. Yu J. Lu G. Zhao W. (2020). Overcoming therapeutic failure in osteosarcoma via Apatinib-encapsulated hydrophobic poly(ester amide) nanoparticles. Biomater. Sci. 8 (21), 5888–5899. 10.1039/d0bm01296c 33001086

[B21] Li Z. Zhang Y. Feng N. (2019). Mesoporous silica nanoparticles: Synthesis, classification, drug loading, pharmacokinetics, biocompatibility, and application in drug delivery. Expert Opin. Drug Deliv. 16 (3), 219–237. 10.1080/17425247.2019.1575806 30686075

[B22] Liang C. C. Park A. Y. Guan J. L. (2007). *In vitro* scratch assay: A convenient and inexpensive method for analysis of cell migration *in vitro* . Nat. Protoc. 2 (2), 329–333. 10.1038/nprot.2007.30 17406593

[B23] Lin Y. S. Wu S. H. Tseng C. T. Hung Y. Chang C. Mou C. Y. (2009). Synthesis of hollow silica nanospheres with a microemulsion as the template. Chem. Commun. (Camb) 2009, 3542–3544. 10.1039/b902681a 19521601

[B24] Liu K. Ren T. Huang Y. Sun K. Bao X. Wang S. (2017). Apatinib promotes autophagy and apoptosis through VEGFR2/STAT3/BCL-2 signaling in osteosarcoma. Cell Death Dis. 8 (8), e3015. 10.1038/cddis.2017.422 28837148 PMC5596600

[B25] Ma X. Zhao Y. (2015). Biomedical applications of supramolecular systems based on host-guest interactions. Chem. Rev. 115 (15), 7794–7839. 10.1021/cr500392w 25415447

[B26] Maluccio M. Covey A. (2012). Recent progress in understanding, diagnosing, and treating hepatocellular carcinoma. CA Cancer J. Clin. 62 (6), 394–399. 10.3322/caac.21161 23070690

[B27] Meng H. Xue M. Xia T. Zhao Y. L. Tamanoi F. Stoddart J. F. (2010). Autonomous *in vitro* anticancer drug release from mesoporous silica nanoparticles by pH-sensitive nanovalves. J. Am. Chem. Soc. 132 (36), 12690–12697. 10.1021/ja104501a 20718462 PMC3116646

[B28] Porta F. Lamers G. E. M. Morrhayim J. Chatzopoulou A. Schaaf M. den Dulk H. (2013). Folic acid-modified mesoporous silica nanoparticles for cellular and nuclear targeted drug delivery. Adv. Healthc. Mater. 2 (2), 281–286. 10.1002/adhm.201200176 23184490

[B29] Qiu X. L. Li Q. L. Zhou Y. Jin X. Y. Qi A. D. Yang Y. W. (2015). Sugar and pH dual-responsive snap-top nanocarriers based on mesoporous silica-coated Fe3O4 magnetic nanoparticles for cargo delivery. Chem. Commun. (Camb) 51 (20), 4237–4240. 10.1039/c4cc10413g 25670321

[B30] She X. Chen L. Velleman L. Li C. Zhu H. He C. (2015). Fabrication of high specificity hollow mesoporous silica nanoparticles assisted by Eudragit for targeted drug delivery. J. Colloid Interface Sci. 445, 151–160. 10.1016/j.jcis.2014.12.053 25617610

[B31] Shen S. Tang H. Zhang X. Ren J. Pang Z. Wang D. (2013). Targeting mesoporous silica-encapsulated gold nanorods for chemo-photothermal therapy with near-infrared radiation. Biomaterials 34 (12), 3150–3158. 10.1016/j.biomaterials.2013.01.051 23369218

[B32] Skrabalak S. E. Chen J. Sun Y. Lu X. Au L. Cobley C. M. (2008). Gold nanocages: Synthesis, properties, and applications. Acc. Chem. Res. 41 (12), 1587–1595. 10.1021/ar800018v 18570442 PMC2645935

[B33] Spraker-Perlman H. L. Barkauskas D. A. Krailo M. D. Meyers P. A. Schwartz C. L. Doski J. (2019). Factors influencing survival after recurrence in osteosarcoma: A report from the children's oncology group. Pediatr. Blood Cancer 66 (1), e27444. 10.1002/pbc.27444 30255612 PMC6249072

[B34] Sun W. Han Y. Li Z. Ge K. Zhang J. (2016). Bone-targeted mesoporous silica nanocarrier anchored by zoledronate for cancer bone metastasis. Langmuir 32 (36), 9237–9244. 10.1021/acs.langmuir.6b02228 27531422

[B35] Teng I. T. Chang Y. J. Wang L. S. Lu H. Y. Wu L. C. Yang C. M. (2013). Phospholipid-functionalized mesoporous silica nanocarriers for selective photodynamic therapy of cancer. Biomaterials 34 (30), 7462–7470. 10.1016/j.biomaterials.2013.06.001 23810081

[B36] Torres Martin de Rosales R. Finucane C. Mather S. J. Blower P. J. (2009). Bifunctional bisphosphonate complexes for the diagnosis and therapy of bone metastases. Chem. Commun. (Camb) 2009, 4847–4849. 10.1039/b908652h PMC7116767 19652801

[B37] Zaheer A. Lenkinski R. E. Mahmood A. Jones A. G. Cantley L. C. Frangioni J. V. (2001). *In vivo* near-infrared fluorescence imaging of osteoblastic activity. Nat. Biotechnol. 19 (12), 1148–1154. 10.1038/nbt1201-1148 11731784

[B38] Zhou X. Cheng X. Feng W. Qiu K. Chen L. Nie W. (2014). Synthesis of hollow mesoporous silica nanoparticles with tunable shell thickness and pore size using amphiphilic block copolymers as core templates. Dalton Trans. 43 (31), 11834–11842. 10.1039/c4dt01138d 24957865

