# Supplementary material for: Controlled-release of apatinib for targeted inhibition of osteosarcoma by supramolecular nanovalve-modified mesoporous silica
Source: Front Bioeng Biotechnol. 2023 Feb 16;11:1135655. doi: 10.3389/fbioe.2023.1135655 (PMC9978000; doi:10.3389/fbioe.2023.1135655)
Supplement: Supplementary file 1 [file DataSheet1.docx]

**Controlled-release of apatinib for targeted inhibition of osteosarcoma by supramolecular nanovalve-modified mesoporous silica**

Xinglong Wang^1,#^, Gongke Li^2,#^, Ke Li^1^, Yu Shi^1^, Wenzheng Lin^1^, Chun Pan^1^, Dandan Li^1^, Hao Chen^1,*^, Jianwei Du^1,*^, Huihui Wang^1,*^

1. Department of Orthopedics, Affiliated Hospital of Yangzhou University. Yangzhou, Jiangsu Province, P. R. China.
2. Department of Critical Care Medicine, Affiliated Hospital of Yangzhou University. Yangzhou, Jiangsu Province, P. R. China.

# These authors contributed equally to this study.

* Corresponding author:

Hao Chen Email: hchen2020@yzu.edu.cn

Jianwei Du Email: doctorduyz@163.com

Huihui Wang Email: wanghh56@yzu.edu.cn


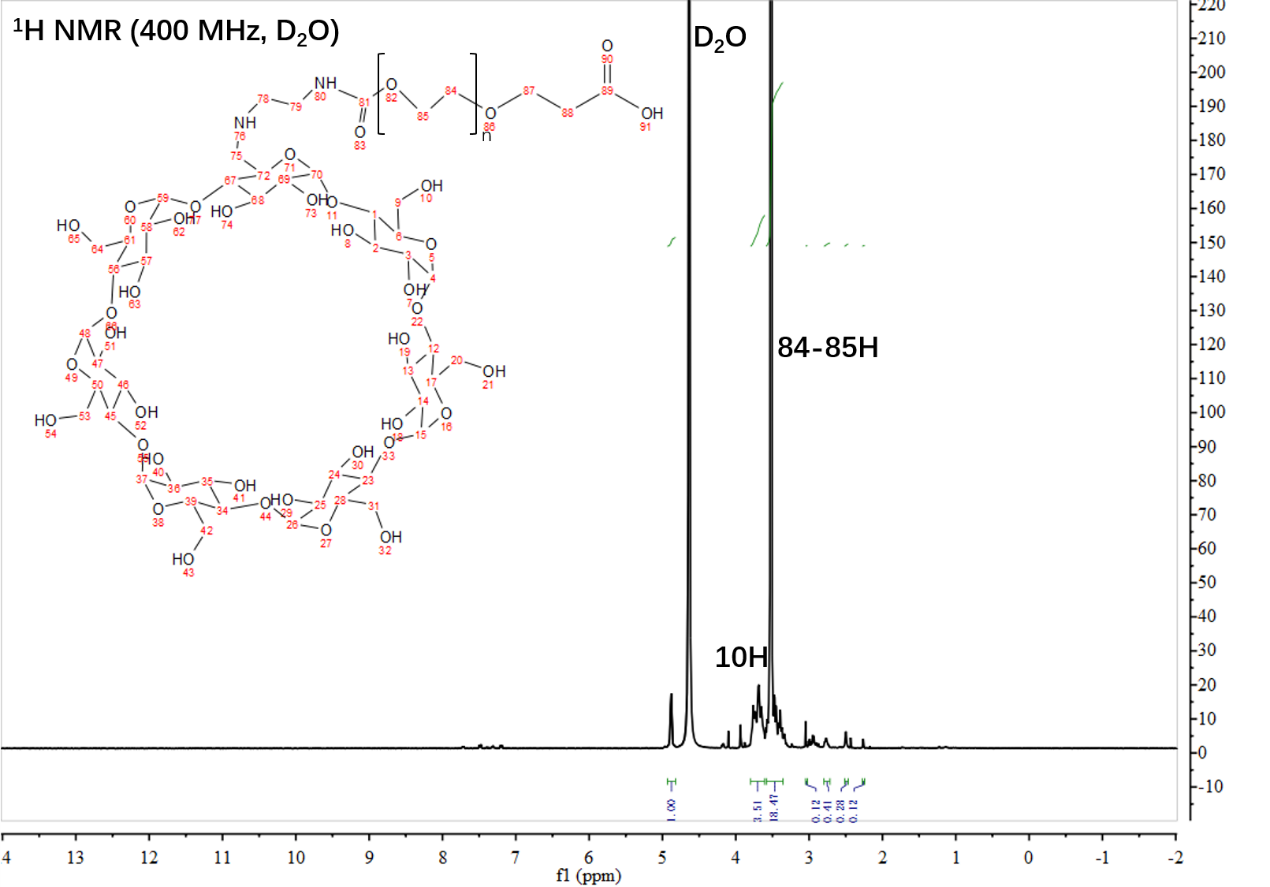


Figure S1. ^1^H-NMR of CD-PEG.


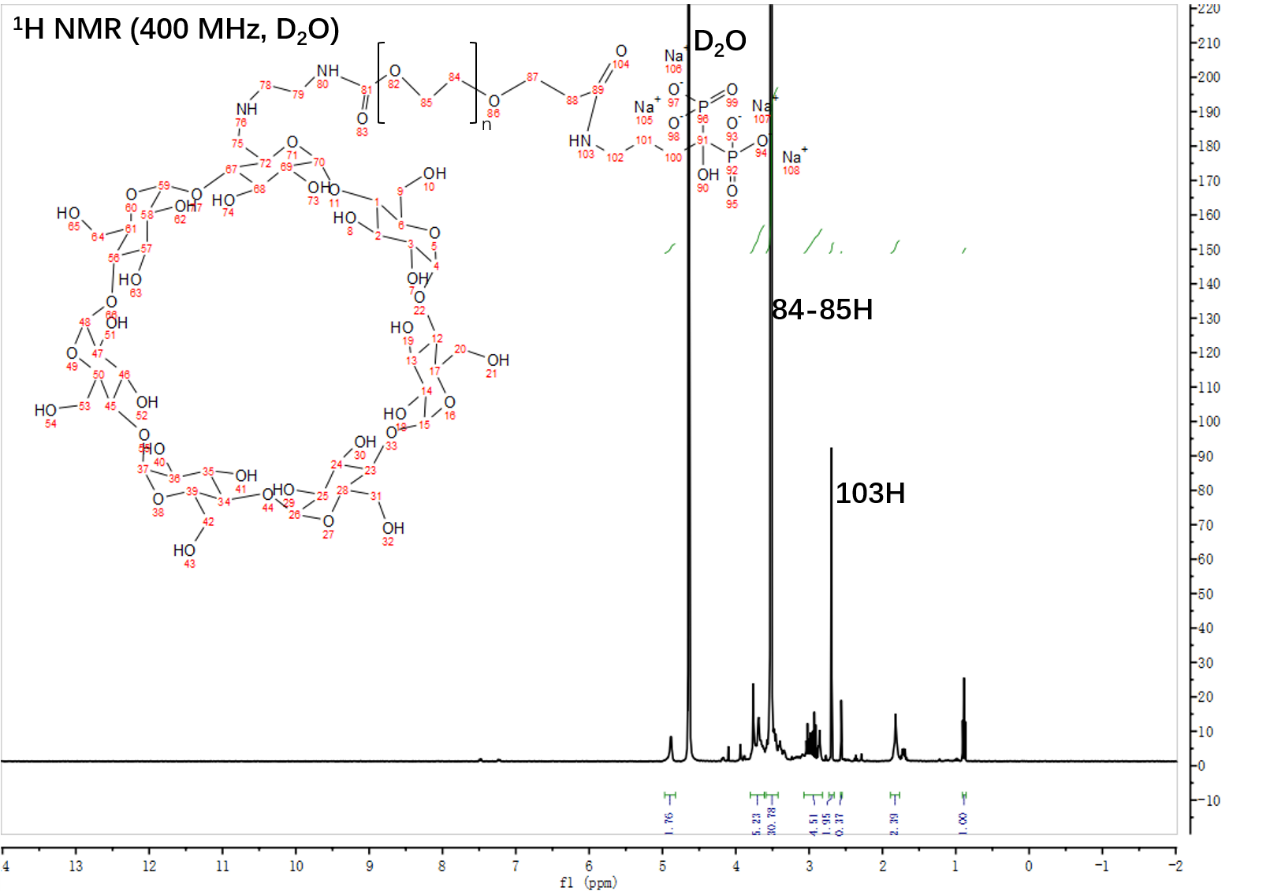


Figure S2. ^1^H-NMR of CD-PEG-ALN.
